# Lebensqualität bei Patienten mit vernarbender und nicht vernarbender Alopezie: eine explorative Querschnittsstudie

**DOI:** 10.1111/ddg.15905_g

**Published:** 2026-03-09

**Authors:** Agathe Franz, Andria Constantinou, Gabriela Engelhardt, Rashmi Singh, Doris Wilborn, Kathrin Hillmann, Sein Schmidt, Ulrike Blume‐Peytavi

**Affiliations:** ^1^ Abteilung für Dermatologie Venerologie und Allergologie Clinical Research Center for Hair and Skin Science Charité – Universitätsmedizin Berlin Körperschaft des öffentlichen Rechts Mitglied der Freien Universität Berlin und der Humboldt‐Universität zu Berlin; ^2^ Klinisches Studienzentrum Berlin Institute of Health an der Charité – Universitätsmedizin Berlin

**Keywords:** Alopezie, Lebensqualität, nicht vernarbende Alopezie, psychologisches Wohlbefinden, Selbstwertgefühl, vernarbende Alopezie, Alopecia, non‐scarring alopecia, psychological well‐being, quality of life, scarring alopecia, self‐esteem

## Abstract

**Hintergrund und Zielsetzung:**

Haarausfallerkrankungen beeinträchtigen die Lebensqualität weit über rein kosmetische Aspekte hinaus. Frühere Untersuchungen berichten meist von milder bis moderater Beeinträchtigung. Ziel dieser Studie war es, das Ausmaß der Lebensqualitätsbeeinträchtigung bei Patienten mit Haarausfall zu erfassen, dabei vernarbende und nicht vernarbende Formen zu vergleichen und mögliche Einflussfaktoren zu analysieren.

**Patienten und Methodik:**

In einer explorativen Querschnittstudie wurden 510 Patienten (281 mit nicht vernarbender und 229 mit vernarbender Alopezie) in der dermatologischen Ambulanz der Charité – Universitätsmedizin Berlin eingeschlossen. Die Lebensqualität wurde anhand des DLQI und ausgewählter PROMIS‐Instrumente erfasst. Zur Auswertung kamen t‐Tests, dreifaktorielle Varianzanalysen mit anschließenden *Post‐hoc*‐Tests sowie Korrelationsanalysen zur Anwendung.

**Ergebnisse:**

Haarausfallerkrankungen können zur Beeinträchtigung der Lebensqualität führen, wobei das psychische Wohlbefinden am stärksten betroffen ist. Die DLQI‐Scores zeigten bei beiden Alopezie‐Typen moderate Einschränkungen. Die PROMIS‐Ergebnisse wiesen bei allen Patienten leichte Angstsymptome auf, bei der nicht vernarbenden Alopezie zusätzlich milde Depressivität. Betroffene mit nicht vernarbender Alopezie berichteten über stärkere psychische Belastung als jene mit vernarbender Alopezie. Patienten beider Gruppen sowie Frauen mit nicht vernarbender Alopezie zeigten teilweise ausgeprägtere Beeinträchtigungen.

**Schlussfolgerungen:**

Die Ergebnisse dieser explorativen Studie deuten darauf hin, dass Haarausfall mit deutlicher psychischer Belastung einhergehen kann. In der Behandlung sollte daher erwogen werden, psychologische Unterstützung in das Behandlungskonzept mit einzubeziehen.

## EINLEITUNG

Alopezie, allgemein als Haarausfall bezeichnet, ist eine vielseitige Erkrankung, deren Ursachen von (auto‐)inflammatorischen Prozessen über hormonelle Dysbalancen und medikamentöse Einflüsse bis hin zu genetischer Veranlagung reichen.

Die Prävalenz variiert stark: Androgenetische Alopezie (AGA) betrifft rund 80% aller Männer europäischer Ethnien und bis zu 40% der europäischen Frauen über 70 Jahre.^1^, ^2^

Haarausfallerkrankungen werden in vernarbende und nicht vernarbende Alopezien unterteilt, wobei erstere seltener sind.^3^, ^4^ Bei der nicht vernarbenden Form bleibt der Haarfollikel erhalten, sodass ein Nachwachsen theoretisch möglich ist.^5^ Bei der vernarbenden Alopezie wird der Haarfollikel durch entzündliche Angriffe auf die Stammzellen in der Wulstregion irreversibel zerstört, wobei die follikulären Strukturen durch fibrotisches Gewebe ersetzt werden und ein Nachwachsen ausgeschlossen ist.^6^

Haarausfall betrifft weit mehr als nur kosmetische Aspekte^7^ und geht häufig mit erheblichen psychischen und sozialen Belastungen einher, etwa vermindertem Selbstwertgefühl und Beeinträchtigungen im sozialen Umfeld.^7^ Die Lebensqualität beschreibt dabei die subjektive Wahrnehmung der physischen, psychischen und sozialen Gesundheit in Bezug auf die Erkrankung.^8^ Zahlreiche Studien untersuchten bereits den Einfluss von Haarausfall auf die Lebensqualität,^9^, ^10^, ^11^, ^12^, ^13^, ^14^, ^15^, ^16^, ^17^, ^18^ doch Vergleiche zwischen vernarbender und nicht vernarbender Form sind bislang selten. Eine Untersuchung an einer kleinen Kohorte von Patientinnen zeigte, dass Frauen mit vernarbender Alopezie deutlich stärkere Beeinträchtigungen ihrer Lebensqualität aufweisen.^19^ Darüber hinaus betonen Forschungsergebnisse zudem die Bedeutung psychiatrischer, psychologischer und psychosozialer Begleitmaßnahmen in der Behandlung von Haarausfall.^20^, ^21^

Unsere Studie hatte zum Ziel, das Ausmaß der Einschränkungen der Lebensqualität bei Alopezie‐Patienten zu erfassen und potenzielle Einflussfaktoren sowie Unterschiede zwischen vernarbender und nicht vernarbender Form zu identifizieren.

## MATERIAL UND METHODIK

Diese explorative Querschnittsstudie wurde gemäß den Richtlinien der Deklaration von Helsinki durchgeführt und von der Ethikkommission der Charité – Universitätsmedizin Berlin (Protokoll‐Nr.: EA2/242/21; Bewilligung am 04.11.2021) genehmigt. Patienten mit vernarbender und nicht vernarbender Alopezie wurden zwischen November 2021 und Juni 2023 in der Haarsprechstunde der Klinik für Dermatologie der Charité – Universitätsmedizin Berlin rekrutiert. Es wurde eine Gelegenheitsstichprobe gebildet, indem alle Patienten, die in diesem Zeitraum die Ambulanz aufsuchten, zur Teilnahme eingeladen wurden. Einschlusskriterien waren deutschsprachige, volljährige Patienten mit einer durch einen Dermatologen bestätigten Haarausfallerkrankung sowie schriftlichem Einverständnis. Zur Minimierung von Verzerrungen durch die Stichprobenauswahl wurden alle in Frage kommenden Teilnehmenden angesprochen.

Erhoben wurden demografische und soziodemografische Merkmale (Alter, Geschlecht, Nationalität, Familienstand, höchster Bildungsabschluss, aktuelle Erwerbstätigkeit, Einkommen, chronische Begleiterkrankungen, Herkunft, Wohnsituation, Kinderzahl) sowie klinische Daten (aktuelle Diagnose, Erkrankungsbeginn, letzte Therapie, betroffene Körperareale). Der Schweregrad der Erkrankung wurde je nach Erkrankungsform mithilfe von Scores erhoben.^22^, ^23^, ^24^, ^25^, ^26^, ^27^

Die Lebensqualität wurde anhand des *Dermatology Life Quality Index* (DLQI) und des *Patient‐Reported Outcomes Measurement Information System* (PROMIS) erhoben. Der DLQI, speziell für dermatologische Erkrankungen entwickelt, umfasst zehn Fragen zu unterschiedlichen Aspekten der Erfahrungen der Patienten in den zurückliegenden 7 Tagen, darunter Symptome, Gefühle, Alltagsaktivitäten, Freizeit, Arbeit beziehungsweise Schule, zwischenmenschliche Beziehungen und Behandlung.^28^ PROMIS, ein allgemeines mehrdimensionales Instrument des NIH Roadmap Network,^29^ erfasst patientenberichtete Endpunkte zu körperlicher, psychischer und sozialer Gesundheit, ebenfalls bezogen auf die letzten 7 Tage. Der Fragebogen *PROMIS‐29 Profile v2.1* ist so aufgebaut, dass er spezifische Bereiche untersucht, die zu einer Beeinträchtigung der Lebensqualität führen können, darunter Erschöpfung, Schlafbeeinträchtigung, körperliche Funktionsfähigkeit, Beeinträchtigung durch Schmerzen, Angst, Depressivität, soziale Isolation sowie die Teilhabe an sozialen Rollen und Aktivitäten.^30^ Vor der Auswertung wurden alle Fragebogen pseudonymisiert.

Angesichts des explorativen Charakters der Studie wurde zu Beginn eine *A‐priori*‐Poweranalyse durchgeführt. Diese ergab, dass bereits für den Nachweis einer mittleren Effektstärke (α = 0,05, *Power* = 80%) in einem Einzelvergleich eine Stichprobengröße von mehr als 50 Teilnehmenden erforderlich wäre. Da jedoch keine formalen Stichprobenberechnungen für die einzelnen Untergruppen vorgenommen und keine fest vorab definierten Hypothesen getestet wurden, strebten wir insgesamt mindestens 500 Teilnehmer an. Dadurch sollten erste Subgruppenanalysen ermöglicht und Hypothesen für weiterführende Untersuchungen generiert werden.

Es wurden deskriptive Analysen für nominale Variablen (zum Beispiel Geschlecht, Diagnose) sowie für metrische Variablen (zum Beispiel Alter, Schweregrad der Erkrankung, Lebensqualität) durchgeführt. Subgruppenanalysen erfolgten jeweils nach Alopezie‐Typ, Geschlecht und Alter. Zur Beschreibung wurden Mittelwerte und Standardabweichungen angegeben. Um Unterschiede zwischen den Patientengruppen zu prüfen, kamen t‐Tests für unabhängige Stichproben zum Einsatz. Zur Untersuchung von Zusammenhängen zwischen Lebensqualität, soziodemografischen Merkmalen und Schweregrad der Erkrankung führten wir Pearson‐Korrelationsanalysen durch. Basierend auf die ermittelten Zusammenhänge wurden die Variablen Alopezie‐Typ, Geschlecht und Alter (eingeteilt in drei Lebensphasen) in eine dreifaktorielle Varianzanalyse (ANOVA) eingebracht. Signifikante Haupteffekte und Interaktionen wurden mittels Bonferroni‐korrigierter Post‐hoc‐Vergleiche weiter untersucht. *p* < 0,05 wurde als statistisch signifikant betrachtet. Die Effektstärken wurden als Cohen's d beziehungsweise partielles Eta‐Quadrat ausgewiesen. Aufgrund der Heterogenität und der unterschiedlichen Größen der Subgruppen erfolgten Vergleiche zwischen Diagnosen ausschließlich deskriptiv. Fehlende Daten wurden in den jeweiligen Analysen ausgeschlossen. Die Datenerhebung und ‐verwaltung erfolgten mit dem elektronischen System REDCap, bereitgestellt an der Charité – Universitätsmedizin Berlin.^31^, ^32^ Die statistische Auswertung wurde mit SPSS (Version 29) durchgeführt.

Weitere Informationen zu Material und Methoden finden sich in den ergänzenden Online‐Informationen.

## ERGEBNISSE

### Studienpopulation

In die Studie wurden 510 Patienten mit Haarausfall eingeschlossen; 26% waren männlich und 74% weiblich. Das mittlere Alter betrug 46,17 ± 16,26 Jahre, die mittlere Dauer des Haarausfalls 78,51 ± 94,77 Monate. Die häufigste Diagnose war Alopecia areata (AA) (42%), gefolgt von frontal fibrosierender Alopezie (FFA) (21%) und Lichen planopilaris (LPP) (16%). Tabelle 1 zeigt eine Übersicht der demografischen und klinischen Merkmale der Studienteilnehmenden.

**TABELLE 1:** Demografische und klinische Daten der Probanden.

		Studienpopulation (n = 510)	Vernarbende Alopezie (n = 229)	Nicht vernarbende Alopezie (n = 281)
**Geschlecht (n = 505)**	Männer	131 (25,9%)	44 (19,4%)	87 (31,3%)
	Frauen	373 (73,9%)	183 (80,6%)	190 (68,3%)
	Divers	1 (0,2%)	0 (0,0%)	1 (0,4%)
**Alter (n = 505)**	Mittelwert (SD)	46,17 (±16,26)	54,88 (±14,44)	39,06 (±14,08)
	< 30	86 (17,0%)	10 (4,4%)	76 (27,3%)
	≥ 30	419 (83,0%)	217 (95,6%)	202 (72,7%)
**Dauer des Haarausfalls (n = 510)**	Mittelwert Dauer in Monaten (SD)	78,51 (± 94,77)	65,80 (± 60,99)	88,86 (± 114,27)
	< 12 Monate	80 (15,7%)	21 (9,2%)	59 (21,0%)
	12 Monate–5 Jahre	225 (44,1%)	117 (51,1%)	108 (38,4%)
	> 5 Jahre	205 (40,2%)	91 (39,7%)	114 (40,6%)

	**Nicht vernarbende Alopezie**						
**Alopecia areata**	n = 214						
SALT‐Score	Mittelwert (SD) (n = 211)				42,08 (± 36,20)		
	Keine: 0%	Leicht: 1–20%	Mäßig: 21–49%		Schwer: 50–94%		Sehr schwer: 95–100%
	9 (4,3%)	85 (40,3%)	34 (16,1%)		50 (23,7%)		33 (15,6%)
**Androgenetische Alopezie** **Männliches Muster**	n = 18						
Hamilton‐Norwood‐ Klassifikation	Mittelwert (SD) (n = 18)				3,28 (± 1,78)		
	I	II	III	III vertex	IV	V	VI
	3 (16,7%)	4 (22,2%)	4 (22,2%)	2 (11,1%)	3 (16,7%)	1 (5,6%)	1 (5,6%)
**Androgenetische Alopezie** **Weibliches Muster**	n = 28						
Ludwig‐Skala	Mittelwert (SD) (n = 26)				1,58 (± 0,64)		
	I		II			III	
	13 (50,0%)		11 (42,3%)			2 (7,7%)	
**Telogenes Effluvium**	n = 17						
**Sonstige nicht vernarbende Alopezien**	n = 3						
	**Vernarbende Alopezie**						
**Lichen planopilaris**	n = 80						
LLPAI‐Score	Mittelwert (SD) (n = 80)				2,60 (± 1,68)		
**Frontal fibrosierende Alopezie**	n = 109						
Rückgang der Haarlinie	Mittelwert (SD) (n = 108)				2,67 (± 1,54)		
	I: < 1 cm	II: < 3 cm		III: < 5 cm		IV: < 7 cm	V: > 7 cm
	7 (6,4%)	60 (55,0%)		34 (31,2%)		4 (3,7%)	4 (3,7%)
**Follikulitis decalvans**	n = 32						
Größte alopezische Fläche	I: < 2 cm		II: < 5 cm			III: ≥ 5 cm	
	7 (21,9%)		15 (46,9%)			10 (31,3%)	
**Perifolliculitis capitis abscedens et suffodiens**	n = 3						
Klassifikation	Stadium 1a		Stadium 1b			Stadium 1c	
	1 (33,3%)		1 (33,3%)			1 (33,3%)	
**Sonstige vernarbende Alopezie**	n = 5						

Abk.: SD, Standardabweichung; SALT, Severity of Alopecia Tool; LLPAI, Lichen Planopilaris Activity Index

### Lebensqualität

Tabelle 2 zeigt die DLQI‐Auswertung. Die PROMIS‐Auswertung ist in Tabelle 3 dargestellt. Die vollständigen Auswertungen der ANOVA‐ und Korrelationsanalysen finden sich in den ergänzenden Online‐Tabellen S4 beziehungsweise S5.

**TABELLE 2:** DLQI‐Werte bei Patienten mit vernarbender und nicht vernarbender Alopezie.

	Gesamte Stichprobe (n = 510)	Nicht vernarbende Alopezie (n = 281)	Vernarbende Alopezie (n = 229)	Vernarbende vs. nicht vernarbende Alopezie	
	*Mittelwert (SD)*	*Mittelwert (SD)*	*Mittelwert (SD)*	*p*	*d*
1. Symptome	1,00 (0,89)	0,85 (0,89)	1,18 (0,85)	< 0,001	−0,39
2. Gefühle	1,48 (1,01)	1,60 (1,03)	1,32 (0,96)	0,001	0,29
3. Alltagsaktivitäten	0,56 (0,89)	0,71 (0,96)	0,39 (0,75)	< 0,001	0,37
4. Kleidung	0,78 (1,07)	1,06 (1,16)	0,43 (0,82)	< 0,001	0,62
5. Soziale Freizeit	0,91 (1,05)	1,18 (1,12)	0,58 (0,85)	< 0,001	0,59
6. Sport	0,55 (0,96)	0,72 (1,07)	0,35 (0,77)	< 0,001	0,39
7. Arbeit und Schule	0,05 (0,23)	0,08 (0,28)	0,02 (0,15)	0,002	0,27
7A. Arbeit und Schule	0,28 (0,56)	0,35 (0,62)	0,19 (0,46)	0,001	0,28
8. Persönliche Beziehungen	0,80 (0,98)	0,98 (1,06)	0,57 (0,82)	< 0,001	0,43
9. Sexuelle Schwierigkeiten	0,71 (1,03)	0,91 (1,11)	0,46 (0,85)	< 0,001	0,45
10. Behandlung	0,64 (0,87)	0,72 (0,93)	0,53 (0,78)	0,014	0,22
DLQI‐Score	7,86 (6,59)	9,32 (7,17)	6,06 (5,27)	< 0,001	0,51

*Abk*.: DLQI, *Dermatology Life Quality Index*; SD, Standardabweichung; p, p‐Wert; d, Effektstärke

**TABELLE 3:** PROMIS‐Werte bei Patienten mit vernarbender und nicht vernarbender Alopezie.

	Gesamte Stichprobe (n = 508)	Nicht vernarbende Alopezie (n = 281)	Vernarbende Alopezie (n = 228)	Vernarbende vs. nicht vernarbende Alopezie	
	*Mittelwert (SD)*	*Mittelwert (SD)*	*Mittelwert (SD)*	*p*	*d*
Emotionale Belastung – Angst	57,44 (9,08)	59,11 (9,53)	55,38 (8,05)	< 0,001	0,42
Emotionale Belastung – Depressivität	53,16 (11,14)	55,44 (11,08)	50,33 (10,57)	< 0,001	0,47
Körperliche Funktionsfähigkeit	46,94 (2,89)	46,94 (3,00)	46,94 (2,75)	0,990	0,00
Erschöpfung	52,28 (10,60)	52,99 (10,90)	51,42 (10,17)	0,098	0,15
Schlafbeeinträchtigung	51,56 (8,37)	51,36 (8,70)	51,81 (7,97)	0,547	−0,05
Beeinträchtigung durch Schmerzen	47,55 (8,50)	46,52 (8,15)	48,82 (8,76)	0,002	−0,27
Teilhabe an sozialen Rollen und Aktivitäten	51,62 (9,39)	50,19 (9,54)	53,39 (8,89)	< 0,001	−0,35
Soziale Isolation	43,64 (9,49)	45,23 (9,88)	41,69 (8,61)	< 0,001	0,38

Abk.: SD, Standardabweichung; p, p‐Wert; d, Effektstärke; PROMIS, Patient‐Reported Outcomes Measurement Information System

Der mittlere DLQI‐Wert aller Teilnehmenden lag bei 7,86 ± 6,59 und weist somit auf eine moderate Beeinträchtigung der Lebensqualität hin. Diese Belastung zeigte sich sowohl bei nicht vernarbender (9,32 ± 7,17) als auch bei vernarbender Alopezie (6,06 ± 5,27). In beiden Gruppen wurden am häufigsten Einschränkungen in Bezug auf Gefühle (DLQI 2) berichtet, gefolgt von Symptomen (DLQI 1) bei der vernarbenden und von sozialer Freizeit (DLQI 5) bei der nicht vernarbenden Alopezie.

In nahezu allen Lebensqualitätsaspekten ergaben sich signifikant höhere Belastungswerte bei Patienten mit nicht vernarbender Alopezie (Tabelle 2). Die ausgeprägtesten Effekte zeigten sich in den Bereichen Kleidung (DLQI 4; *p* < 0,001, d = 0,62), soziale Freizeit (DLQI 5; *p* < 0,001, d = 0,59), persönliche Beziehungen (DLQI 8; *p* < 0,001, d = 0,43) und sexuelle Schwierigkeiten (Item 9; *p* < 0,001, d = 0,45). Bei Symptomen (DLQI 1) berichteten hingegen Betroffene mit vernarbender Alopezie eine höhere Belastung (*p* = 0,001; η^2^η = 0,02). Die ANOVA unter Kontrolle von Alter und Geschlecht bestätigte diesen Unterschied (Tabelle S4 im Online‐Supplement).

Betrachtet man die einzelnen Diagnosen, so erreichten nur Betroffene mit FFA einen DLQI‐Wert < 6 (geringer Einfluss), während alle anderen Diagnosen eine moderate Einschränkung zeigten (Tabelle S1 im Online‐Supplement).

Sowohl Patienten mit vernarbender Alopezie (T‐Wert = 55,38 ± 8,05) als auch mit nicht vernarbender Alopezie (T‐Wert = 59,11 ± 9,53) wiesen eine leichte Beeinträchtigung hinsichtlich Angst auf. Darüber hinaus zeigten Personen mit nicht vernarbender Alopezie leichte Depressivität (T‐Wert = 55,44 ± 11,08). Auffällig war, dass die Angst‐ (*p* < 0,001; d = 0,42) und Depressionswerte (*p* < 0,001; d = 0,47) bei nicht vernarbender Alopezie höher lagen als bei der vernarbenden Form (Tabelle 3). Die ANOVA ergab keinen signifikanten Haupteffekt des Alopezie‐Typs (Tabelle S4 im Online‐Supplement).

Auf Diagnosenebene war die Angst bei allen Erkrankungen außer bei FD erhöht: Bei AA, FFA und LPP in leichtem Ausmaß, bei AGA und TE in moderatem Ausmaß. Leichte Depressivität trat zudem bei AA, AGA und TE auf; bei AGA wurden darüber hinaus leichte Erschöpfung und Schlafbeeinträchtigung dokumentiert (Tabelle S1 im Online‐Supplement).

### Geschlechtsspezifische Analysen

In der ANOVA zeigte sich ein signifikanter Haupteffekt des Geschlechts, wobei Frauen höhere Angstwerte angaben als Männer (*p* = 0,002; η_p_^2^ = 0,02). Dieser Unterschied war bei Frauen mit nicht vernarbender Alopezie besonders ausgeprägt und zeigte sich in einer signifikanten Interaktion zwischen Geschlecht und Alopezie‐Typ (*p* = 0,021; η_p_^2^ = 0,011). Auch in Bezug auf Gefühle (DLQI 2; *p* = 0,017; η_p_^2^ = 0,01) sowie hinsichtlich Schwierigkeiten in persönlichen Beziehungen (DLQI 8; *p* = 0,032; η_p_^2^ = 0,01) ergab sich jeweils eine signifikante Interaktion zwischen Geschlecht und Alopezie‐Typ. In beiden Bereichen berichteten Frauen mit nicht vernarbender Alopezie höhere Beeinträchtigungen.

### Altersbezogene Analysen

Die ANOVA zeigte, dass jüngere und mittelalte Erwachsene signifikant höhere DLQI‐Werte aufwiesen als ältere Erwachsene (*p* = 0,021; η_p_^2^ = 0,02). *Post‐hoc*‐Vergleiche bestätigten, dass sowohl die junge als auch die mittlere Altersgruppe sich jeweils deutlich von der älteren Gruppe unterschieden (*p* < 0,001). Bei Patienten mit nicht vernarbender Alopezie nahm sowohl die Ausprägung der Depressivität (r = –0,22; *p* < 0,001) als auch das Ausmaß sozialer Isolation (r = –0,19; *p* = 0,002) mit steigendem Alter ab. Darüber hinaus berichteten jüngere und mittelalte Erwachsene höhere Angstwerte (*p* = 0,026; η_p_^2^ = 0,02) sowie eine stärkere emotionale Belastung in Bezug auf Gefühle (DLQI 2; *p* = 0,005; η_p_^2^ = 0,02) als ältere Erwachsene. Auch hier zeigten *Post‐hoc*‐Tests signifikante Unterschiede zwischen beiden jüngeren Gruppen und den älteren Teilnehmenden für Angst (*p* < 0,001 und *p* = 0,043) sowie für die emotionale Belastung (*p* = 0,003 und *p* = 0,001).

### Weitere Einflussfaktoren

Bei Patienten mit nicht vernarbender Alopezie zeigte sich kein Zusammenhang zwischen kürzlich erfolgter Behandlung und den Lebensqualitäts‐Parametern. Bei vernarbender Alopezie ging jedoch das Ausbleiben einer Therapie in den letzten drei Monaten mit erhöhter sozialer Isolation (r = 0,15; *p* = 0,028) und höherem DLQI‐Wert (r = –0,17; *p* = 0,010) einher.

Weder bei vernarbender noch bei nicht vernarbender Alopezie bestand ein Zusammenhang zwischen Beginn der Erkrankung und den Lebensqualitäts‐Parametern.

Darüber hinaus fanden sich Assoziationen, wenn der Haarausfall neben der Kopfhaut auch andere Körperregionen betraf. Die stärksten Zusammenhänge zeigten sich bei nicht vernarbender Alopezie und Wimpernausfall: Hier war die Teilhabe an sozialen Rollen und Aktivitäten eingeschränkt (r = –0,13; p = 0,036), soziale Isolation stärker ausgeprägt (r = 0,17; *p* = 0,004), Depressivität höher (r = 0,12; *p* = 0,043) und der DLQI‐Wert erhöht (r = 0,15; *p* = 0,012).

Für alle Erkrankungen außer FD und AGA korrelierte der Schweregrad der Erkrankung mit höheren DLQI‐Werten (*p* < 0,01). Bei schwerem Verlauf der AA zeigte sich diese Korrelation zudem für alle PROMIS‐Domänen mit Ausnahme von Angst, Erschöpfung und Schlafbeeinträchtigung. Bei Patienten mit FFA ging eine höhere Krankheitsschwere mit erhöhter Erschöpfung einher (r = 0,21; *p* = 0,031).

Zwischen allen PROMIS‐Domänen und den DLQI‐Werten bestanden signifikante Korrelationen.

## DISKUSSION

Unsere Studie verdeutlicht den ausgeprägten Einfluss von Haarausfall (sowohl nicht vernarbend als auch vernarbend) auf die Lebensqualität der Patienten und zeigt insbesondere eine signifikante Beeinträchtigung des psychischen Wohlbefindens. Diese Beobachtung bestätigt frühere Untersuchungen.^9^, ^10^, ^11^, ^12^ Die PROMIS‐Auswertung verdeutlicht zudem erhöhte Angstwerte in allen Diagnosen, mit Ausnahme der FD.

Hervorzuheben ist, dass psychische Beeinträchtigungen die Lebensqualität maßgeblich beeinflussen: Sowohl bei nicht vernarbender als auch bei vernarbender Alopezie zeigen sich ausgeprägte Angst‐ und emotionale Belastungen, wobei die nicht vernarbende Form stärkere Auswirkungen aufweist. Zusätzlich trat Depressivität vor allem in dieser Gruppe gehäuft auf, was die höhere psychische Beanspruchung bei nicht vernarbender Alopezie unterstreicht. Im Gegensatz dazu war die körperliche Funktionsfähigkeit bei vernarbender Alopezie stärker eingeschränkt. Dies ist auf typische Symptome der vernarbenden Form wie Pruritus, Brennen und Dysästhesien zurückzuführen, die zu körperlichen Beschwerden und Schmerzen beitragen können.^33^

Im Gegensatz zu unserer Studie zeigte eine ausschließlich weibliche Kohorte von Katoulis et al. eine stärkere Beeinträchtigung bei Patientinnen mit vernarbender Alopezie. Die DLQI‐Werte für nicht vernarbende Alopezie fielen in beiden Studien nahezu identisch aus (9,4 ± 3,45 vs. 9,32 ± 7,17).^19^ Dagegen wichen die DLQI‐Werte für vernarbende Alopezie deutlicher ab (12,3 ± 3,37 vs. 6,06 ± 5,27). Hier ist zu bemerken, dass das Durchschnittsalter der Patienten mit nicht vernarbender Alopezie in beiden Untersuchungen gleich war, während die Teilnehmer mit vernarbender Alopezie in unserer Studie älter waren.

Insgesamt scheinen Frauen stärker betroffen zu sein als Männer. Dies zeigt sich besonders deutlich bei Frauen mit nicht vernarbender Alopezie, die über erhöhte Angstwerte und größere Schwierigkeiten in persönlichen Beziehungen berichten. Frühere Studien konnten einen direkten Zusammenhang zwischen Beeinträchtigung durch Haarausfall und Geschlecht nur schwer nachweisen.^34^, ^35^ Allgemein nahm die Beeinträchtigung mit zunehmendem Alter ab, wobei die Literatur hierzu uneinheitliche Ergebnisse aufweist.^10^, ^11^, ^13^, ^15^, ^34^, ^35^ Der Zusammenhang zwischen Geschlecht, Alter und Alopezie ist komplex und vermutlich kulturabhängig: Für jüngere Generationen und insbesondere für Frauen hat Haar eine hohe ästhetische und identitätsstiftende Bedeutung, was das Selbstwertgefühl stärker belastet. Ältere Personen können sich eher auf den Haarausfall einstellen oder greifen gegebenenfalls stärker auf Hilfsmittel wie Perücken zurück.

Obwohl, ähnlich wie in anderen Studien,^15^, ^16^, ^17^ kein signifikanter Zusammenhang zwischen Beeinträchtigung und Erkrankungsbeginn nachgewiesen wurde, erweist sich der Einfluss der Therapie als bedeutsam. Bei nicht vernarbender Alopezie zeigte sich keine signifikante Korrelation zwischen dem Vorhandensein einer Therapie und der Lebensqualität, wobei anzumerken ist, dass medikamentöse Behandlungen in Deutschland überwiegend nicht erstattungsfähig sind und der Zugang zu neueren, potenziell wirksameren Therapieoptionen dadurch erschwert wird. Im Gegensatz dazu war bei vernarbender Alopezie das Ausbleiben einer Therapie mit insgesamt höherer Beeinträchtigung (DLQI) und stärkerer sozialer Isolation verbunden, was darauf hindeutet, dass eine wirksame Therapie die Lebensqualität positiv beeinflussen kann.

Ebenso sollte berücksichtigt werden, dass der Schweregrad der Erkrankung die Beeinträchtigung maßgeblich beeinflussen kann. Für AA, FFA und LPP zeigte sich eine zumindest teilweise Korrelation zwischen Schweregrad und DLQI‐ beziehungsweise PROMIS‐Werten. Bei AGA (weibliches und männliches Muster) sowie FD ließ sich hingegen keine solche Assoziation feststellen, vermutlich bedingt durch kleinere Patientengruppen bei diesen Krankheitsbildern. Da die jeweils verwendeten Schweregradskalen zwischen den Erkrankungen variieren, sind die Werte nicht direkt vergleichbar.

Die in allen PROMIS‐Domänen und im DLQI beobachteten Korrelationen unterstreichen die Reliabilität beider Instrumente. Sie unterscheiden sich jedoch von spezifischen Fragebögen zum Haarausfall, die einen längeren Erhebungszeitraum berücksichtigen, was angesichts der variablen Krankheitsaktivität und möglicher Schübe sinnvoller sein könnte. Untersuchungen, die verschiedene Zeitfenster verglichen haben, zeigen, dass über längere Zeiträume deutlich höhere Belastungswerte berichtet werden.^16^ Die Ausweitung des Erfassungszeitraums im DLQI führte ebenfalls zu höheren Beeinträchtigungswerten,^18^ und betont damit die Bedeutung des Zeitfaktors bei der Lebensqualitätsbewertung von Haarausfallerkrankungen. Der DLQI betrachtet Dermatologie spezifische Aspekte, insbesondere solche, die das äußere Erscheinungsbild betreffen, kann jedoch nicht alle Besonderheiten von Haarausfallerkrankungen abbilden. Das multidimensionale PROMIS‐Instrument liefert einen umfassenderen Überblick über die Lebensqualität und ermöglicht Vergleiche mit anderen Krankheitsbildern.

Nur wenige Studien haben PROMIS zur Erfassung der dermatologischen Lebensqualität genutzt. So untersuchten Esaa et al. in einer longitudinalen Studie verschiedene Hauterkrankungen hinsichtlich Beeinträchtigung durch Schmerzen, Depressivität und Angst.^36^ Die Ergebnisse zeigten für Akne, atopische Dermatitis und Psoriasis, die von erstatteten Behandlungsoptionen profitieren, keine signifikanten Beeinträchtigungen bei Angst, Depressivität oder Schmerzbeeinträchtigung. Bei der Hidradenitis suppurativa dagegen wurden deutliche Belastungen durch Angst und Schmerz erfasst. Der DLQI wurde bereits in zahlreichen Studien zur Bewertung der Lebensqualität eingesetzt, unter anderem bei Vitiligo, Psoriasis und Akne, wobei die Ausprägung der Beeinträchtigungen unterschiedlich ausfiel.^37^, ^38^, ^39^ Im Vergleich zu diesen Krankheitsbildern weisen unsere DLQI‐Werte für AA und LPP stärkere Beeinträchtigungen auf, während die Werte für FFA dazwischen liegen. Unsere Daten belegen zudem eine ausgeprägte Angstbelastung sowohl bei vernarbender als auch bei nicht vernarbender Alopezie und eine stärkere Depressivität bei der nicht vernarbenden Form. Dieses Ergebnis verdeutlicht die besondere psychische Belastung durch Haarausfall angesichts der eingeschränkten Erstattungsmöglichkeiten für Therapien.

Diese Studie weist mehrere Limitationen auf. Zum einen verwendeten wir eine Gelegenheits‐Stichprobe von Patienten, die wegen Haarausfall eine Behandlung suchten, was einen Selektionsbias zur Folge haben und die Übertragbarkeit auf die allgemeine Population einschränken kann. Das Fehlen einer Kontrollgruppe reduziert zudem die Interpretierbarkeit unserer Ergebnisse.

Unsere Daten basieren auf einem einmalig eingesetzten Fragebogen mit Bezug auf einen kurzen Zeitraum, sodass keine Längsschnittanalysen möglich sind. Weder der DLQI noch PROMIS sind spezifisch für Alopezie validiert; der DLQI wurde jedoch in zahlreichen Haarausfallstudien eingesetzt^9^, ^10^, ^11^, ^12^, ^13^, ^14^, ^15^, ^16^, ^17^, ^18^, ^19^ und vermittelt einen breiten Eindruck, wie sich Alopezie auf die Lebensqualität auswirkt. Darüber hinaus umfasst die Studie mehrere heterogene Subgruppen für die unterschiedlichen Alopezieformen, die meist nur kleine Patientengruppen sowie variable Alters‐ und Geschlechterverteilungen aufweisen. Auch dies schränkt die Generalisierbarkeit unserer Befunde ein. Trotz dieser Limitationen liefert die explorative Untersuchung erste Einblicke in die Belastung durch verschiedene Alopezieformen auf die Lebensqualität der Betroffenen.

Um die Generalisierbarkeit unserer Ergebnisse zu beurteilen, wurden Faktoren wie Stichprobenwahl, Studiendesign und demographische Daten der Patienten berücksichtigt. Wir rekrutierten eine umfangreiche Kohorte von Patienten aus unterschiedlichen Regionen Deutschlands, schlossen beide Geschlechter, ein breites Altersspektrum sowie verschiedene Formen des Haarausfalls ein, um die externe Validität der Befunde zu stärken.

Angesichts des explorativen Charakters dieser Querschnittsstudie sollten unsere Ergebnisse als Ausgangspunkt für die Konzeption größer angelegter Untersuchungen betrachtet werden. Zukünftige Studien könnten größere, nach Alter und Geschlecht abgeglichene Subgruppen einschließen, um das Ausmaß der Lebensqualitätsbeeinträchtigung bei Alopezie präziser zu erfassen. Wiederholte Messungen über längere Zeiträume würden dabei helfen, die Beeinträchtigung genauer abzubilden. Der Einsatz oder die Entwicklung validierter, speziell auf Haarausfallerkrankungen zugeschnittener Fragebögen sowie die Einbindung von Kontrollgruppen könnten zudem die Studienmethodik weiter stärken.

Zusammenfassend deuten unsere Ergebnisse darauf hin, dass die psychische Belastung durch Alopezie beachtlich sein kann und daher verstärkte Berücksichtigung verdient. Unsere Studie widerlegt die Auffassung, Haarausfallerkrankungen seien rein kosmetische oder Lifestyle‐Erkrankungen, indem sie ihr potenzielles Ausmaß an Lebensqualitätseinschränkungen, insbesondere im emotionalen Bereich, aufzeigt. Diese Ergebnisse legen nahe, dass psychologische Unterstützungsangebote als Bestandteil der Behandlung von Haarausfall sinnvoll sein könnten, auch wenn weitere Studien erforderlich sind, um diesen Ansatz zu validieren.

## DANKSAGUNG

Wir danken allen, die zur Datenerhebung beigetragen haben, insbesondere Dr. med. T. Tomova‐Simitchieva, Dr. med. D. A. Lintzeri und K. M. H. Alqurashi.

Open access Veröffentlichung ermöglicht und organisiert durch Projekt DEAL.

## FÖRDERUNG

Agathe Franz hat ein Promotionsstipendium der Charité – Universitätsmedizin Berlin erhalten.

## INTERESSENKONFLIKT

U. B.‐P. war Referentin und/oder Beraterin und/oder Prüferin und/oder hat Forschungsgelder von AbbVie, Adaran, Almirall, Amryt, Bayer, Boots Healthcare, Cantabria Labs, Cassiopeia, CeraVe, Dermocosmétique Vichy, Eli Lilly, FomF, Galderma Laboratorium GmbH, GSK, Infectopharm, Johnson & Johnson, Laboratoires Bailleul, Legacy, Leo Pharma, Novartis, Pfizer, Pierre Fabre, Sanofi Regeneron und Sun Pharmaceutical erhalten. Alle anderen Autoren geben an, keinen Interessenkonflikt zu haben.

## Supporting information



Supplementary information
